# Did Cold Resistance Influence the Success of the Halobiont Darkling Beetle *Centorus rufipes* (Coleoptera, Tenebrionidae) During the Pleistocene?

**DOI:** 10.3390/insects17020204

**Published:** 2026-02-14

**Authors:** Roman Yu. Dudko, Anna A. Gurina, Arcady V. Alfimov, Natalia I. Agrikolyanskaya, Ilya I. Lyubechanskii, Ekaterina N. Meshcheryakova, Sergei V. Reshetnikov, Andrei A. Legalov, Daniil I. Berman

**Affiliations:** 1 Institute of Systematics and Ecology of Animals, Siberian Branch of the Russian Academy of Sciences, Novosibirsk 630091, Russia; rdudko@mail.ru (R.Y.D.); natylnik@mail.ru (N.I.A.); lubech@gmail.com (I.I.L.); reshetnikov-art@yandex.ru (S.V.R.); fossilweevils@gmail.com (A.A.L.); 2 Institute of Biology, Ecology and Natural Resources, Kemerovo State University, Kemerovo 650000, Russia; 3 Institute of Biological Problems of the North, Far Eastern Branch of the Russian Academy of Sciences, Magadan 685000, Russia; arcalfimov@gmail.com (A.V.A.); kameshky@mail.ru (E.N.M.); dberman@mail.ru (D.I.B.); 4 Department of Natural Sciences, Novosibirsk State University, Novosibirsk 630090, Russia; 5 Department of Forestry and Landscape Construction, Tomsk State University, Tomsk 634050, Russia; 6 Biology Department, Samarkand State Pedagogical Institute, Samarkand 140102, Uzbekistan

**Keywords:** cold resistance, supercooling point, low lethal temperature, West Siberian Plain, late Pleistocene

## Abstract

*Centorus rufipes* (Gebler, 1833) is the only tenebrionid beetle commonly found in late Pleistocene deposits of southern western Siberia. It is assumed that the reasons for its success during the Last Glacial Maximum could have been its cold resistance and/or the relatively mild conditions of its habitat, the shores of salt lakes. The cold resistance parameters of *C. rufipes* and their overwintering conditions were studied near Kusgan Lake (Novosibirsk Oblast, Russia). Adults and larvae of this species used a supercooling mechanism to protect themselves from sub-zero temperatures and did not tolerate freezing, just like other steppe species of Tenebrionidae. The supercooling point (*SCP*) for most of the individuals was around −31 °C. Measurements of low lethal temperatures (*LLT*) showed that 50% of individuals died after 2 days of exposure to −27 °C. The measured *SCP* and *LLT* were at least 5 °C lower than darkling beetle species from the Chuya Depression of the Altai, which is known for its extreme winter temperatures. Thus, the hypothesis of increased cold resistance of *C. rufipes* was confirmed. No warming effect of its salt lakeside habitats was detected.

## Simple Summary

We studied the cold resistance of the darkling beetle *Centorus rufipes*, the only species of darkling beetle abundant in the late Pleistocene mammoth steppes of western Siberia. In contrast, darkling beetles are one of the leading groups in the modern analog of the mammoth steppes. Insufficient cold resistance has been suggested as the reason for the low prevalence of darkling beetles in the Pleistocene. However, this is insufficiently substantiated without understanding the peculiarities of *C. rufipes*. Therefore, the following hypotheses were tested: *C. rufipes* either has high cold resistance that allows it to survive at low temperatures, or it successfully selects microhabitats with relatively mild conditions. The supercooling point for most of the sampled beetles was around −31 °C, and the death temperature of 50% of individuals was approximately −27 °C. These values were at least 5 °C lower than darkling beetle species from the Chuya Depression of the Altai Mountains, known for its extreme winter temperatures. Thus, the hypothesis that increased cold resistance of *C. rufipes* was important for its success in the Pleistocene was supported.

## 1. Introduction

Periglacial tundra–steppe landscapes, also known as “mammoth steppes”, occupied much of Northern Asia at the end of the late Pleistocene [1]. However, paleoenvironmental reconstructions of this biome are largely contradictory, and debate continues regarding the degree of correspondence (similarities and differences) between the mammoth steppes and their modern analogs [2,3,4]. Identifying significant differences between the two and interpreting them is crucial for understanding the conditions of the late Pleistocene.

Insects, and in particular, Coleoptera, are one of the most important groups used in paleoreconstructions of the Quaternary period [5,6]. Thus, a rich and highly specific beetle fauna, including more than 400 species, has been identified from the late Pleistocene deposits of the southern West Siberian Plain [7,8,9]. This fauna differs sharply from the modern one of this region, which has sufficiently mild winters (average January air temperature is −20 °C), but is relatively similar to the modern fauna of the intermountain depressions of Southern Siberia and Northern Mongolia (average January air temperature drops to −35 °C) [8,9,10,11]. However, an unexpected feature of the Pleistocene fauna is the very poor representation of darkling beetles (Tenebrionidae), although at present, this is one of the most abundant groups in steppe and desert habitats of Southern Siberia [12,13]. A surprising exception is the darkling beetle *Centorus rufipes* (Gebler, 1833), found in several late Pleistocene sites of the West Siberian Plain and the Cis-Urals, where it was sometimes abundant [8,9,14,15].

The limiting factor for some species of this family might be cold resistance, i.e., the ability of individuals to survive in low (negative) temperatures, especially during long-term exposure. The body fluids of poikilotherm animals, which are not cold-resistant, freeze at sub-zero temperatures, and the resulting ice crystals irreversibly damage cells. Cold-resistant animals either have a lower freezing point (which prevents ice formation) or have developed the ability to withstand freezing using a variety of physicochemical mechanisms. The first is characterized by the supercooling point (SCP), the temperature at which the supercooled liquid begins to freeze; the second is indicated by the lower lethal temperature (LLT), which shows resistance to prolonged exposure to cold [16,17].

Unfortunately, the study of cold resistance in darkling beetles remains scarce. There are members of the darkling beetle family that are supercooling, such as the desert species *Microdera punctipennis* Kaszab, 1967) [18], and also others that are freeze-resistant, such as the dendrobiont *Upis ceramboides* (Linnaeus, 1758) [19].

The limited cold resistance of darkling beetles might be inferred based on the distribution of species that currently inhabit the extracontinental climate of the southeastern Altai (*Anatolica dashidorzsi* Kaszab, 1965, *Penthicus altaicus* (Gebler, 1829) and *Bioramix picipes* (Gebler, 1833)). These species cannot withstand long-term cooling below −22 °C, which suggests that the reason for the low diversity of darkling beetles in the late Pleistocene of Siberia is their insufficient cold resistance [20]. However, to confirm this assumption, it is necessary to understand how *C. rufipes* survived the conditions of the late Pleistocene. This species, unlike most tenebrionids, is a halobiont that lives near the shores of salt lakes, like other species of the genus *Centorus* Mulsant, 1854 [21].

The aim of our work was to clarify the reasons for the success of *C. rufipes* in the late Pleistocene in south-western Siberia. Two hypotheses were put forward: (1) the species has higher cold resistance than other steppe species of darkling beetles; and (2) the proximity of salt lakes moderates conditions while the species is overwintering. It should also be noted that confirmation of at least one of these hypotheses would also provide a verification of the previous assumption about the influence of minimum winter temperatures on the past and present distribution of steppe darkling beetles in Northern Asia [20].

To achieve this goal, the following tasks were set.

1. Find overwintering sites of *C. rufipes*, determine overwintering stages, and collect material in the pre-winter season to determine cold resistance.
2. Measure the cold resistance of overwintering stages.
3. Estimate the temperature conditions of overwintering in the modern range of *C. rufipes* using climate reference books and databases.
4. Measure soil temperatures and snow depth at the *C. rufipes* collection site at different distances from the water.
5. Compare the cold resistance of *C. rufipes* and other darkling beetle species with the temperature conditions at their overwintering sites.

## 2. Materials and Methods

### 2.1. Taxonomical Remarks, Distribution and Ecology of Centorus rufipes

**Figure 1:**
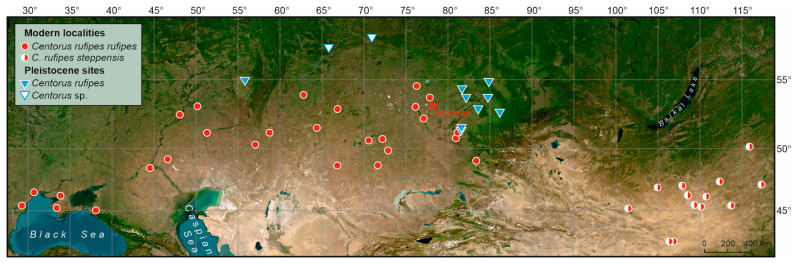
Modern and late Pleistocene distribution of *Centorus rufipes* according to [8,9,15,21,24] and collections of Siberian Zoological Muzeum, Institute of Systematics and Ecology of Animals (Novosibirsk) and Zoological Institute (Saint-Petersburg). A red arrow indicates Kusgan Lake, from which the material for the current study was collected. The map background of the study region derived from the Esri in ArcGIS Online (version of June 2025) (https://www.arcgis.com/apps/mapviewer/index.html?layers=10df2279f9684e4a9f6a7f08febac2a9 accessed on 1 September 2025).

**Figure 2:**
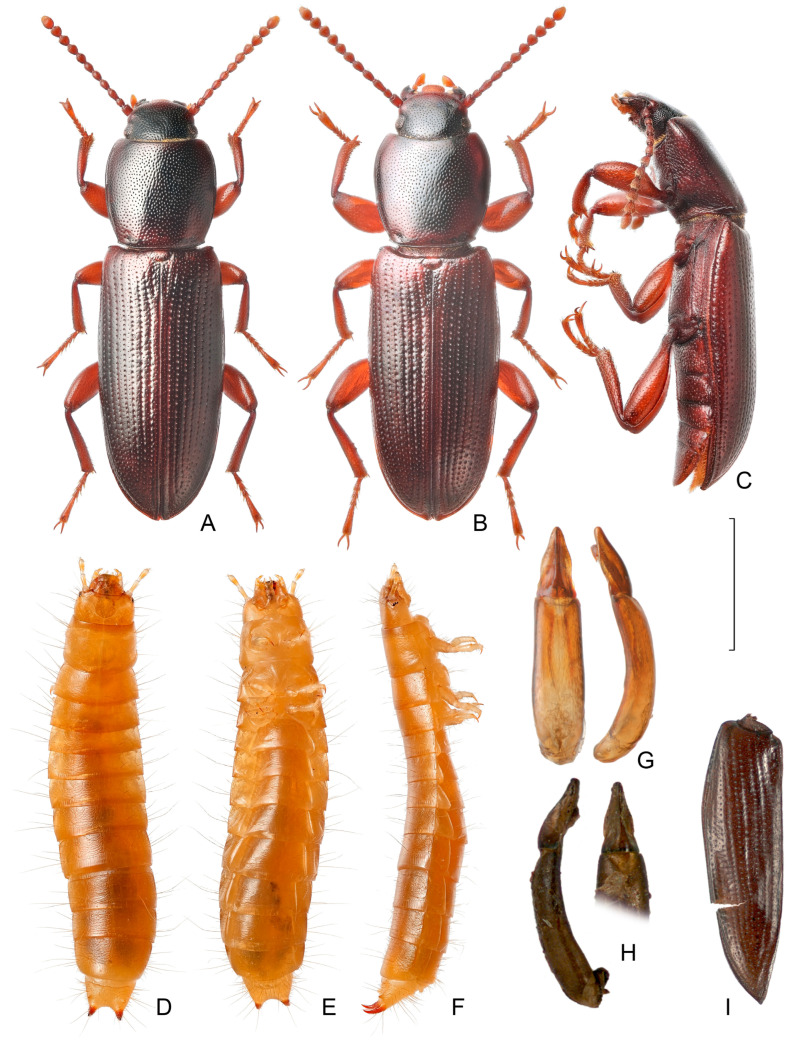
Images of *Centorus rufipes* from Kusgan Lake (**A**–**G**) and subfossil late Pleistocene remains from Suzun-1 site (**H**,**I**) [9]. (**A**–**C**) These represent the habitus of adult female (**A**) and male (**B**,**C**), dorsal and lateral views; (**D**–**F**)—habitus of older larva, dorsal, ventral and lateral views; (**G**,**H**)—aedeagus, dorsal and lateral views; (**I**)—left elytron. Scale bar is 2 mm (**A**–**F**,**I**) and 1 mm (**G**,**H**).

*Centorus rufipes* (Gebler, 1833) belongs to the tribe Belopini, subfamily Lagriinae [22]. The interpretation of the species remains controversial. The nominate *C. r. rufipes* is distributed in the steppe zone of Eurasia from the Black Sea estuaries in the west to the foothills of Altai in the east (Figure 1 and Figure 2). A similar taxon, *C. rufipes steppensis* (Kaszab, 1964), was described as a distinct species from eastern Mongolia [23]. In the revision of the tribe Belopini, Skopin [21] noted that these taxa, according to all diagnostic features, including the structure of parameres, have intermediate specimens, and therefore he considered *Belopus steppensis* as an eastern subspecies of *B. rufipes*. Later, Medvedev in his key to Mongolian darkling beetles [24] again listed it as an independent species without any comments. The species status of the taxon remained in the Palearctic catalog [22]. However, we consider Skopin’s interpretation to be more justified. In any case, these taxa cannot be distinguished in the Quaternary material; therefore, the distribution of the species in eastern Mongolia and Transbaikalia is accepted.

*Centorus* cf. *rufipes* was found in several late Pleistocene sites (MIS 3 and MIS 2) in the southeast of the West Siberian Plain (Figure 2H,I), and is also known from the Southern Cis-Urals [8,9,15]. The northernmost records of the genus *Centorus* (probably *C. rufipes*) from the Malkovo and Skorodum-95 sites (57–58° N) were reported by Kiselev [25] and Zinoviev [26]. Late Pleistocene sites of *Centorus* spp. are located significantly north and east of the modern range of *C. r. rufipes* (Figure 1).

There is virtually no information in the literature about the ecology of the species. It is only known that it inhabits predominantly loose, periodically moist saline soils, with stocky grassy vegetation of the subfamily Chenopodioideae [21].

### 2.2. Study Area

The area of study was chosen to be on the northeastern edge of the modern range of *C. r. rufipes*, in close proximity to the Pleistocene records (Figure 1). Kusgan Lake is located at the junction of the Baraba forest–steppe and the Kulunda depression of the steppe zone of the West Siberian Plain. The topography is characterized by numerous 50–60 m-high ridges; in the depressions there are chains of large and small endorheic lakes, often with salty or bitter-salty water. Southern and ordinary chernozem soils predominate in the area, but in the lowlands, large areas of solonchak, solonetz and saline chernozem soils are common [27].

### 2.3. Collection Localities and Metods

**Figure 3:**
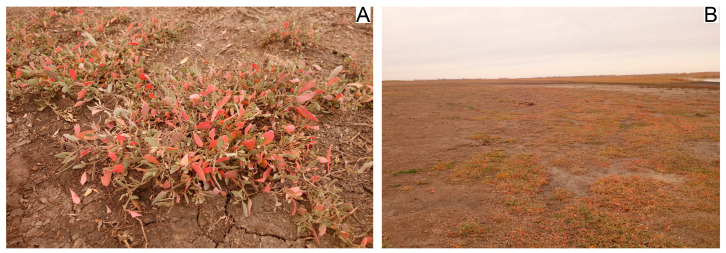
Host plant *Atriplex verrucifera* (**A**) and habitat (**B**) of *Centorus rufipes* on the shore of Lake Kusgan in October 2023.

Insects were collected in the Karasuk district of Novosibirsk Oblast (Russia) on the southern shore of the brackish Lake Kusgan (53.7337° N, 77.8569° E). The salinity of the lake is 1.83 g/L, and mainly consists of sodium sulphate, sodium chloride and sodium bicarbonate [28]. The beetles were found only on a small, slightly elevated (by a few decimeters) area adjacent to the lake; surrounded on both sides by small hollows, which are flooded in early spring, forming a peninsula measuring 70 m × 150 m. Due to soil salinity, the vegetation here was poor and was represented only by rare clumps of the saltbush *Atriplex verrucifera* M. Bieb., 1808 up to 15 cm high (Figure 3).

Material was collected on 11–12 October 2023 and included 126 adults and 10 larvae of *C. rufipes*. All of them were found in leaf litter and soil at a depth of no more than 5 cm under *A. verrucifera* bushes. The collected beetles and larvae were kept in containers with soil for 26 days in a refrigerator at a temperature of 5–6 °C.

At the beginning of November, darkling beetles were delivered to the Institute of Biological Problems of the North, Far Eastern Branch of the Russian Academy of Sciences (Magadan), which has the necessary equipment for measuring of cold resistance.

### 2.4. Measuring Cold Resistance

Two standard parameters of cold resistance by *C. rufipes* were studied: (1) supercooling point (*SCP*), and (2) lower lethal temperature (*LLT*).

Before the start of the experiments, 10–21 *C. rufipes* adults were placed into plastic containers (50 mL) filled to two-thirds with substrate taken from the animals’ habitat. Before measuring cold resistance, beetles were acclimated according to scheme previously used on other beetles of this family [20]: 14 days at 5 °C, 7 days at 3 °C, 20 days at 1 °C, 14 days at −1 °C, 7 days at −2 °C, 3 days at −3 °C. The scheme simulated the seasonal course of soil temperatures in darkling beetle habitats. After acclimation, the beetles were kept under “mild” overwintering conditions (21 days at −5 °C).

At temperatures of 10 and 5 °C, insects were kept in cooling thermostats TSO-1/80 (Smolenskoe SKTB SPU, LLC, Smolensk, Russia), followed by acclimation (from 3 °C to −3 °C), and for mild overwintering, they were kept in a Binder MK 53 programmable test chamber (Binder, GmbH, Tuttlingen, Germany). To determine *SCP* and *LLT*, programmable test chambers WT 64/75 (Weiss Umwelttechnik, GmbH, Reiskirchen–Lindenstruth, Germany) were used.

*SCP* was measured thermoelectrically using a manganin–constantan thermocouple (wire diameter 0.12 mm). The signal from the thermocouples was converted through a DC amplifier using the LA-TK5 analog-to-digital converter (Rudnev-Shilyaev, LLC, Moscow, Russia) and recorded by a computer. Thermocouples were mounted in pairs on a cork base, and placed in a copper box measuring 5 cm × 8 cm × 8 cm with a wall thickness of 2 mm for uniform cooling. The box was placed in a WT 64/75 test chamber for programmed cooling.

To measure *SCP*, the beetles that were used were under mild overwintering conditions. Individuals were attached to the manganin–constantan thermocouple junction with Vaseline. A total of 31 adults and 8 larvae were tested.

Since the number of specimens available was limited, the choice of *LLT* temperatures was based on the obtained *SCP* values, which provided an indication of the survival of darkling beetles after exposure to different temperatures. For *LLT* determination, adults were exposed to −25 °C, −30 °C, and −40 °C; for larvae, *LLT* determination was not carried out. The darkling beetles were kept at each of the tested temperatures for 2 days, after which they were heated successively to 0, +1, +5 °C, and were kept at each stage for one day, then transferred to +20 °C. Individuals that restored typical behavioral reactions at the end of two weeks of observation were considered survivors.

To control possible temperature fluctuations in the chambers, calibrated temperature loggers DS1922L (Elin, STL, Moscow, Russia) were placed next to the containers with insects. Their individual error at 0 °C was ±0.2 °C.

The methods and equipment used have been described in detail previously [20,29,30].

### 2.5. Study of Overwintering Conditions

Data loggers TR-2L (Engineering Technologies, LLC, Moscow, Russia) were used to determine the temperature conditions of *C. rufipes* overwintering at the field site, as well as the influence of the lake on soil temperature. Four data loggers were installed in the soil at a depth of 1 cm (at distances of 30, 70, 150 and 380 m from the lake), three loggers at a depth of 10 cm (30, 70 and 150 m from the lake) and one logger at a depth of 40 cm (30 m from the lake). All data loggers were installed on 17 November 2023 (before temperatures crossed 0 °C) and removed on 8 April 2024 (after soil thaw), temperature was recorded every 4 h.

Snow depth was measured on 3 March 2024, at 15 sites along three transects perpendicular to the lakeshore. Ten measurements were taken in each site every 2 m along a line parallel to the shore. The first transect passed through the place where insects were collected and loggers were installed, the second and third ones were located 300 and 700 m to the east.

Information on air temperature in winter 2023–2024 were obtained from the Karasuk weather station, located 10 km from the insect collection site [31]. Long-term series of climate characteristics and data from several weather stations in the winter of 2023–2024 were obtained from the websites [11,31,32]. Data from climate reference books [33,34,35,36,37,38,39,40] were used to describe the average long-term conditions of the cold season in the central (northern Kazakhstan, southern West Siberian Plain) and eastern (eastern Mongolia, Transbaikalia) parts of the range of *C. rufipes*. The significantly milder winter climate in the western part of the range (Black Sea regions) was not considered.

Minimum soil temperatures at a depth of 3 cm were calculated using the nomograms of Shulgin [41] based on data on minimum annual air temperatures and snow depth.

### 2.6. Statistical Analysis

Statistical parameters for *SCP* and snow depth (mean, standard deviation, and median) were calculated using the PAST 4.11 software. The cartographic base with space images was obtained in the SAS.Planet 200606.10075 software.

## 3. Results

### 3.1. Cold Resistance

Under mild overwintering conditions (−5 °C), the adults and larvae were in a supercooled state; they remained soft to the touch but were motionless.

**Figure 4:**
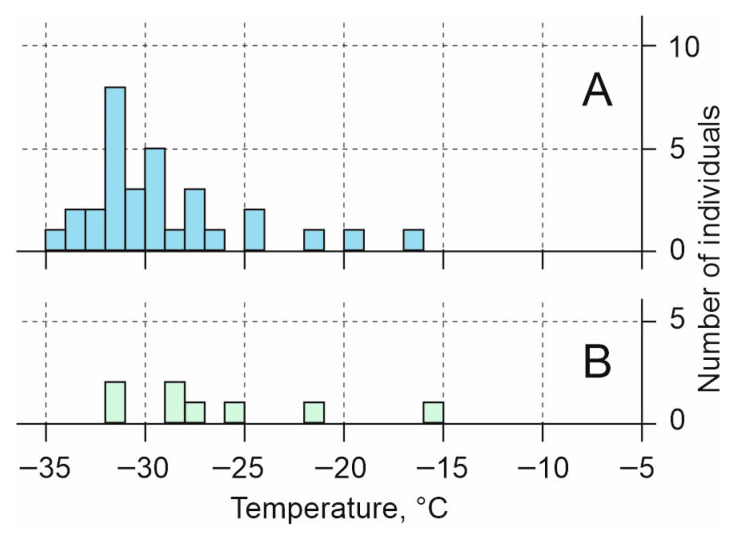
Histogram of *SCP* (°C) of *Centorus rufipes*. (**A**)—adults, (**B**)—larvae.

The range of *SCP* values for adults was from −16.5 °C to −34.0 °C, with a mean of −29.0 °C ± 0.7 °C, and a median of −30 °C, but the main data array was in the range from −26 to −34 °C, with a peak around −31 °C (Figure 4A).

The *SCP* values of larvae were distributed in the range from −15 °C to −31 °C, with a mean of −26.1 ± 1.9 °C, and a median of −28 °C, with five of the eight individuals having *SCP* values that were low for this species, from −28 °C to −31 °C (Figure 4B).

After a mild overwintering exposure, 95% of adults survived. Also, the majority of beetles in the sample (63%) revived after exposure to −25 °C, but only 19% of animals survived exposure to −30 °C (Table 1).

All adults and larvae died after cooling below the minimum *SCP*, indicating that they were intolerant to freezing and could only overwinter in a supercooled state (Table 1). Thus, the *LLT* at which 50% of adult individuals survive was below −25 °C (estimated from −27 °C to −28 °C).

### 3.2. Climatic Conditions near Kusgan Lake in the 2023–2024 Season

**Figure 5:**
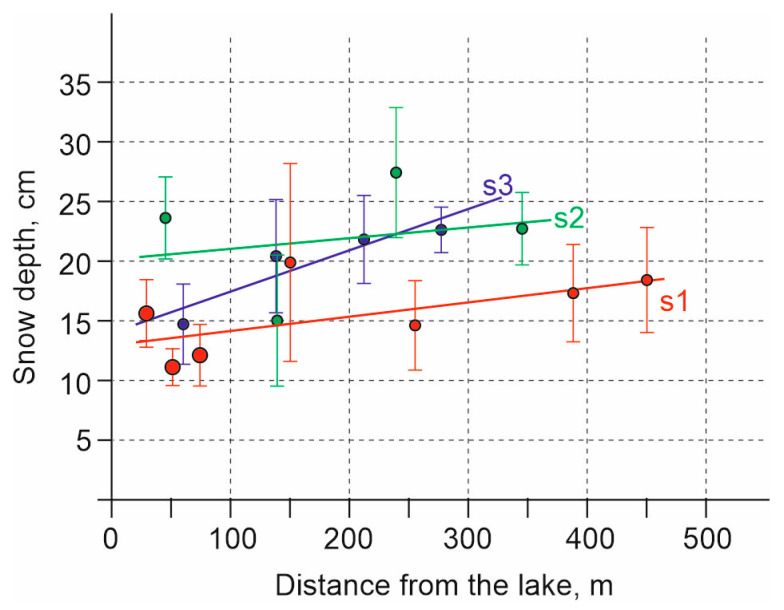
Dependence of snow depth on distance from lakeshore measured on transects s1–s3 on 3 March 2024, on the distance to the Kusgan Lake. Vertical lines indicate ±S.D.

Snow depth measurements taken on 3 March 2024 showed uneven distribution, with depths ranging from 7 to 38 cm (18 cm on average) in the southern outskirts of Kusgan Lake, and from 9 to 19 cm (13 cm on average) directly in areas inhabited by *C. rufipes*. At the same time, a direct dependence of snow depth on the distance to the lake was revealed on all three transects. On average, the snow becomes 1–2 cm deeper with a distance of 100 m from the lake (Figure 5).

**Figure 6:**
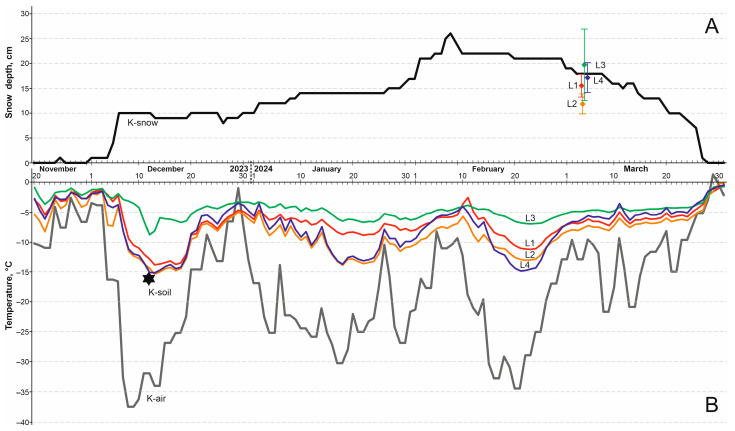
Snow depth (**A**) and temperatures (**B**) near the collecting sites of *C. rufipes* during 2023–2024 season. K-snow, K-air—snow depth and daily minimum of air temperature in Karasuk weather station, and K-soil—the point of annual minimum soil temperature at a depth of 3 cm calculated from these data. L1, L2, L3 and L4—daily minimum temperature of 1 cm depth soil by data loggers, installed at 30 m, 70 m, 150 m and 380 m from the Kusgan Lake (**B**), and snow depth ± standard deviation measured on 3 March over these loggers (**A**).

At four sites located at distances from 30 to 380 m from the shore of Kusgan Lake, the lowest soil temperatures were recorded in mid-December, after air temperatures dropped to −37.4 °C (Figure 6). In the coldest sites, with little snow (L2) and remote from the lake with medium-height snow (L4), the annual minima at a depth of 1 cm were −15.4 °C and −15.6 °C, respectively. The warmest area was the snowiest site (L3), where the temperature at a depth of 1 cm did not fall below −8.8 °C (Figure 6). Minimum annual temperatures at a depth of 10 cm ranged from −13.6 °C in the coldest site (L2) to −7.7 °C in the warmest one (L3).

The temperature conditions for the overwintering of insects at the place of their collection in the 2023–2024 season were close to the long-term average. Thus, the absolute minimum air temperatures at the Karasuk weather station in the winter of 2023–2024 were close to the climate norm (−37.4 °C and −37.0 °C, respectively). The maximum, average and minimum snow depths according to the snow survey data we conducted (38, 18 and 7 cm) were very close to the average long-term values in the vicinity of the Karasuk weather station—41, 22 and 5 cm.

### 3.3. Variation in Overwintering Conditions of Centorus rufipes in Recent Decades

At the Karasuk weather station, the closest to the *C. rufipes* collection site, no observations of the soil temperature under the snow were carried out. To analyze long-term variation, data from the Slavgorod weather station, located 80 km to the southeast, were used; the air temperature and snow depth at this station were close to those in the insect collection area.

**Figure 7:**
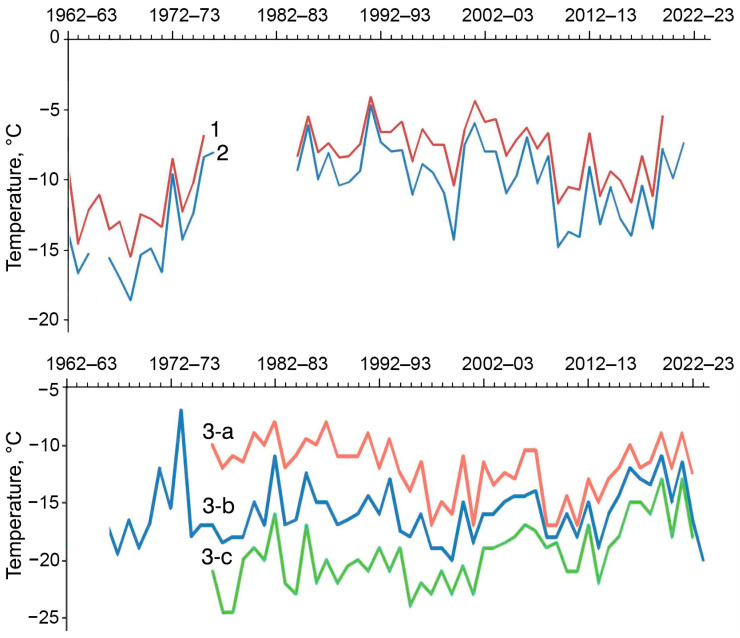
Long-term variation in annual minimum soil temperatures at the Slavgorod weather station at different depth: 1—40 cm, 2—20 cm, 3—3 cm in high-snow (a), middle-snow (b) and low-snow (c) sites.

At the Slavgorod weather station, the minimum soil temperatures at depths of 40 and 20 cm (which are significantly greater than the overwintering depth of *C. rufipes*) did not fall below −17.5 °C and −18.6 °C, respectively, over more than 50 years of observations (Figure 7). The lowest temperatures were recorded in the 1960s, and the highest in the 1980s and the very beginning of the 2000s.

Both adults and larvae of *C. rufipes* overwinter in the upper 5 cm of soil, where temperatures are not measured at weather stations. The annual minimum temperatures at a depth of 3 cm calculated using Shulgin’s nomograms [41], in contrast to those measured at 20 and 40 cm, did not show a pronounced long-term trend (Figure 7). According to calculations, over almost 50 years of observations, in areas with a lot of snow, soil temperatures at a depth of 3 cm did not fall below −17 °C and below −25 °C in areas with little snow. With an average snow depth, the absolute minimum temperature is estimated at −20 °C.

### 3.4. Climate Conditions Within the Range of Centorus rufipes

**Figure 8:**
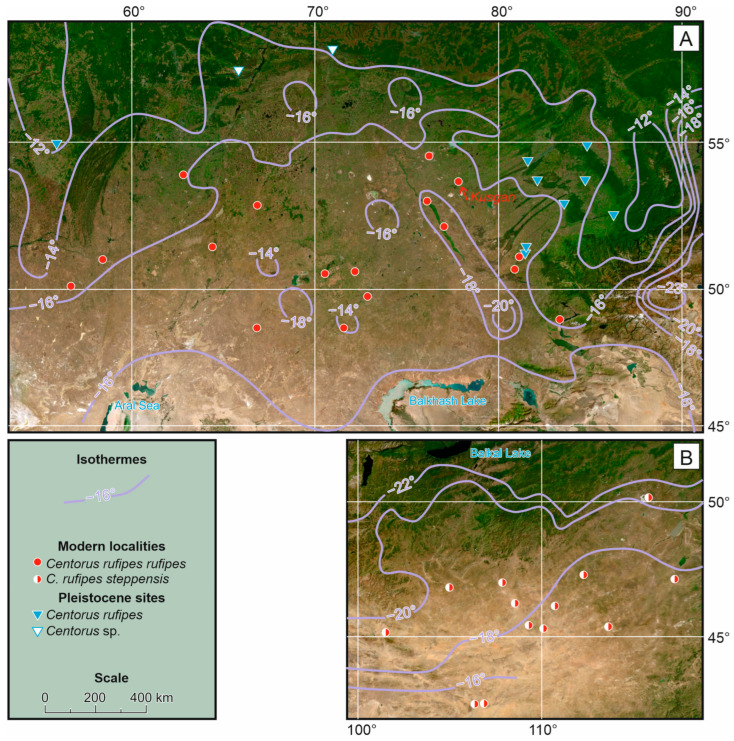
Isotherms of annual minimum soil temperatures at 3 cm depth within the central (**A**) and eastern (**B**) parts of the range of *Centorus rufipes*. The isotherms were calculated using the Shulgin’s nomograms [37] based on data on minimum annual air temperatures and snow depth [33,34,35,36,37,38,39,40]. The map background of the study region derived from the Esri in ArcGIS Online (version of June 2025) (https://www.arcgis.com/apps/mapviewer/index.html?layers=10df2279f9684e4a9f6a7f08febac2a9 accessed on 1 September 2025).

The Asian anticyclone centered over the mountainous regions of eastern Kazakhstan and western Mongolia is the main climate-forming factor in both the central and eastern parts of the *C. rufipes* range in winter. In the central range, air temperatures decrease from southwest to northeast, and snow depth decreases from north to south. The temperatures of the upper centimeters of soil formed by the above-mentioned factors decrease from west to east, right up to the foothills of Altai and Western Sayan, where the increase in snow depth again creates milder overwintering conditions (Figure 8A). As a result, in the central part of the range of *C. rufipes*, the lowest average long-term values at a depth of 3 cm (about −18 °C) were calculated for the dry steppe zone in northeastern Kazakhstan. In the area where *C. rufipes* was collected, the temperatures mentioned (from −16 to −17 °C) were close to the average for the entire central part of its range.

A similar calculation of mean long-term minimum soil temperatures at a depth of 3 cm in eastern Mongolia showed that their gradient in the range of *C. rufipes steppensis* was directed from northwest to southeast, and the temperatures themselves varied from −20 °C to −16 °C (Figure 8B). In the mountainous regions of Mongolia, dividing the two parts of the range, minimum soil temperatures, as shown previously [20], fall below −20 °C (up to −22 °C) in most cases.

## 4. Discussion

### 4.1. Cold Resistance

Based on our results, adults and larvae of *C. rufipes* used a single strategy of protection from the cold: they did not tolerate freezing and overwintered in a supercooled state. The same strategy is widespread in the darkling beetle family, as it has been found in five other species living in the Russian Altai—*Anatolica dashidorzsi*, *Penthicus altaicus*, *Bioramix picipes*, *Pedinus femoralis* (Linnaeus, 1767) and *Blaps lethifera* (Marsham, 1802) [20], and also in *Microdera punctipennis*, which inhabits the Gurbantünggüt Desert in Xinjiang [18].

The *SCP* for adult of *C. rufipes* was the lowest in the darkling beetle family, on average 3–14 °C lower than those obtained for other species [16,18].

The temperature at which approximately half of the adult *C. rufipes* can withstand long-term exposure was approximately −27 °C or −28 °C, which is slightly warmer than the mean (−29 °C) and median (−30 °C) *SCP* values. Available data allow us to estimate the threshold temperature for overwintering at about −30 °C, since less than 20% of individuals survive with prolonged exposure to lower temperatures.

Based on *LLT* values, *C. rufipes* is the most cold-resistant species of darkling beetles, overwintering in a supercooled state. For *Anatolica dashidorzsi*, *Penthicus altaicus* and *Bioramix picipes*, the 50% mortality threshold lies in the temperature range of −20 to −25 °C, and for *Blaps lethifera*, in the range of −10 to −15 °C [20]. Darkling beetles, *Microdera punctipennis*, have an average *SCP* of −18.7 °C, with a minimum of around −20 °C, which is significantly higher than that of species from the Chuya Depression of Russian Altai. A special case is the dendrobiont *Upis ceramboides* from Yakutia, which is very taxonomically distant from the steppe-living darkling beetles; it overwinters in a frozen state and tolerates temperatures below −83 °C [19].

To understand the significance of the obtained *SCP* and *LLT* values for *Centorus rufipes*, they were compared with similar indicators of insects that overwinter in the soil and also do not tolerate freezing, inhabiting the continental regions of northeastern Russia. Winter climate conditions here are close to those in northeastern Yakutia, the generally recognized “cold pole” of the Northern Hemisphere.

In adults of 12 species of ants (Formicidae) in northeastern Russia, the *SCP* range varies widely, from −17 °C in the most cold-sensitive species to −44 °C in the most resistant; in larvae, *SCP* values reach −46 °C. Long-term lower lethal temperatures (*LLT*) for most species are 3–5° higher than the mean *SCP*. Larvae and adults of eight species of click beetles (Elateridae) have average *SCP* values ranging from −26 to −31 °C, with the *LLT* of 50% mortality also 5 °C higher. The mealybug *Arctorthezia cataphracta* (Shaw, 1794) (Hemiptera, Coccoidea) overwinters at all post-embryonic stages; its mean *SCP* is about −32 °C, anda half of the individuals die within 24 h at a temperature of −27 °C. In 10 species of true locusts (Orthoptera), the *SCP* of overwintering eggs reaches −36 °C [30]. Thus, the cold resistance of *C. rufipes* is close to that of the most cold-resistant insect species that overwinter in a supercooling state in northeastern Asia.

### 4.2. Temperatures During Overwintering of Centorus rufipes and the Influence of Salt Lakes

The average long-term minimum temperatures of the upper soil layers throughout the range of *C. rufipes* were in the range of −14 to −20 °C, which was significantly higher than the threshold temperature tolerated by this species (about −30 °C). In the south of the West Siberian Plain, in the habitats of *C. rufipes*, soil temperatures have not reached this value over the past 50 years; even in areas with little snow, the temperature has not dropped below −25 °C. Thus, within its modern range, this species is not limited by winter temperatures.

The stenotopic nature of the genus *Centorus*, which is confined to saline soils on the shores of lakes, raises the question of the beneficial influence of the habitat, allowing it to live in colder climates, for example, during the Last Glacial Maximum. The warming effect of lakes and other bodies of water during the winter is well-known [42], but it is far from clear whether it is strong enough to affect the upper centimeters of soils in the overwintering sites of *C. rufipes*. In addition, salt lakes and their depressions indirectly affect soil temperature due to the uneven distribution of snow, which depends on the relief, microtopography and the degree of vegetation development.

The data loggers recorded the same minimum temperatures of the upper soil layers both in the habitats of *C. rufipes* (logger L2, 70 m from the lake) and at a significant distance (logger L4, 380 m). These data indicate the absence of a pronounced warming effect of the lake in our experiment and are consistent with the experiments of Voronina [43] and Srebryanskaya [44], showing that solonetz soils are 1–2 °C warmer than ordinary chernozems only at a depth of 20–40 cm, primarily due to high moisture. However, the issue of the influence of the habitat requires additional comprehensive study, and at the moment, it cannot be considered resolved.

### 4.3. On the Reasons of the Success of Centorus rufipes in the Pleistocene

In the south of the West Siberian Plain at the end of the Late Pleistocene (marine isotope stages 3 and 2) there were open periglacial tundra–steppe, forest–steppe or steppe landscapes that are absent today. They were formed in conditions of cold and dry climate with widespread permafrost [1,45]. Note that due to the influence of permafrost, both the heat availability and the duration of the warm period in the soil should have been reduced, but not the minimum temperatures in the first centimeters of soil, which, however, are associated with *C. rufipes* larvae at all stages of development, and adults during overwintering. It follows that the appearance or intensification of permafrost should not have affected the distribution of the species, due to its cold resistance.

Reconstruction of quantitative climate parameters for non-analogous communities, especially in winter, is extremely difficult [15]. Reliable interpretations of the LGM time have been obtained from Lake Aksor deposits, northeastern Kazakhstan, 100 km southeast of Pavlodar. The average annual temperature dropped by no less than 13 °C compared to today’s (+4 °C), the average annual precipitation was less than 100 mm, and winters were snowless with strong winds [46,47]. According to other reconstructions [1,45], the average temperatures of the coldest month in the LGM should have been from −32 °C to −35 °C. At present, the closest climatic analogs are the continental regions of Yakutia, where the average January temperatures are close to above-mentioned. The minimum air temperatures here drop to −46 °C or even to −50 °C [48]. At such air temperatures and a snow depth of 3–5 cm, the temperature in the upper soil layers should have dropped to −26 °C or even to −28 °C [41]. Thus, of all the darkling beetle species whose cold resistance is known, only *Centorus* could have withstood the conditions under LGM climate reconstructions [20].

Previously, it was shown that there is no “cold resistance reserve” (i.e., the difference between cold resistance and minimum temperatures in overwintering sites) in steppe darkling beetles of Southern Siberia, especially in *Anatolica dashidorzsi* and *Penthicus altaicus* living in the Chuya Depression [20]. It was assumed that successful overwintering of such species is possible only in the warmest microhabitats, under shrubs and tillering nodes of large cereals. On the contrary, *C. rufipes rufipes* now ranges in a region with noticeably milder winters, where background temperatures of the upper soil layers are 5–9 °C higher than in the Chuya Depression of Altai. At the same time, *C. rufipes* turned out to be significantly more cold-resistant, its *LLT* values being 5–8 °C lower than those of species from the Chuya Depression [20]. This “cold resistance reserve” clearly exceeds the level necessary for living under modern climate conditions, suggesting that this species was well-adapted to the conditions of the late Pleistocene. It is likely that the success of the species in LGM conditions is due to its relatively high cold resistance.

The numerous records of *C. rufipes* and its high abundance in some late Pleistocene sites suggest a widespread distribution of this species in the past. At present, the sporadic distribution of the species suggests a reduction in its former range, i.e., *C. rufipes* can be considered a Pleistocene relic. A similar reduction in range and abundance is characteristic of many Pleistocene species of beetles, including those associated with saline habitats: *Poecilus (Derus) ravus* (Lutschnik, 1922), *P. (D.) major* (Motschulsky, 1844), *Harpalus amputatus* Say, 1830 (Carabidae), *Aclypea bicarinata* (Gebler, 1830), *A. sericea* (Zoubkoff, 1833) (Silphidae) [7,9,14,15,49,50]. The success of these species in the Pleistocene once again raises the question of the warming influence of salt lakes.

## 5. Conclusions

The halobiont darkling beetle *Centorus rufipes* overwinters as adults and larvae of various ages in the upper layers of soil near salt lakes. Both adults and larvae of this species used a supercooling mechanism to protect themselves from sub-zero temperatures and did not tolerate freezing, just like other steppe species of darkling beetles. *SCP* values of adults ranged from −16.5 to −34.0 °C with a peak at −31 °C. Survival during prolonged cooling was close to freezing temperature; more than 50% of individuals died at temperatures below −27 °C, and about 80% at −30 °C. The latter value can be considered as the lower threshold temperature tolerated by this species.

*Centorus rufipes* has sufficient “cold resistance” to tolerate variation found throughout its range. Average long-term minimum temperatures of the upper soil layers in different regions varied from −14 to −20 °C, and in the south of the West Siberian Plain in the habitats of this species over the past 50 years, soil temperatures did not drop below −25 °C, even in areas with little snow.

A comparison with other steppe species of darkling beetles showed that *C. rufipes* was the most cold-resistant, and its *LLT* values were 5–8 °C lower than other tenebrionids from the coldest steppe regions of North–Central Asia. This “excessive” cold resistance of *C. rufipes* for its modern range indicates that, in the past, it could overwinter under much colder conditions. Thus, the success of the species in the Pleistocene, in particular during the Last Glacial Maximum, was associated with its high cold resistance. The hypothesis of a warming effect of salt lakes on the habitats of *C. rufipes* was not confirmed. However, there was insufficient evidence to completely reject this hypothesis due to the complex direct and indirect influence of lakes and salinization on the environment.

## Figures and Tables

**Table 1:** Survival of *Centorus rufipes*, adults when exposed to low temperatures for two days.

Temperature, °C	Number of Individuals	Survivors, %
−5	20	95
−25	19	63
−30	21	19
−40	15	0

## Data Availability

All data needed to evaluate the conclusions in the paper are present in the paper. Additional data related to this paper may be requested from the corresponding author.

## Abbreviations

The following abbreviations are used in this manuscript:

LGM	last glacial maximum
*LLT*	low lethal temperature
*SCP*	supercooling point

## References

[1] Frenzel B., Frenzel B., Pécsi M., Velichko A.A. (1992). Maximum cooling of the last glaciation (about 20 000 to 18000 y. BP). Atlas of Paleoclimates and Paleoenvironments of the Northern Hemisphere, Late Pleistocene and Holocene.

[2] Yurtsev B.A. (2001). The Pleistocene “Tundra-Steppe” and the productivity paradox: Thelandscape approach. Quat. Sci. Rev..

[3] Zimov S.A., Zimov N.S., Tikhonov A.N., Chapin F.S. (2012). Mammoth steppe: A high-productivity phenomenon. Quat. Sci. Rev..

[4] Chytrý M., Horsák M., Danihelka J., Ermakov N., German D.A., Hájek M., Hájková P., Kočí M., Kubešová S., Lustyk P. (2019). A modern analogue of the Pleistocene steppe-tundra ecosystem in southern Siberia. Boreas.

[5] Elias S.A. (1994). Quaternary Insects and Their Environments.

[6] Kuzmina S.A. (2017). Macroentomology analysis: Methods, opportunities, and examples of reconstructions of paleoclimatic andpaleoenvironmental conditions in the Quaternary of the northeastern Siberia. Contemp. Probl. Ecol..

[7] Zinovyev E. (2011). Sub-fossil beetle assemblages associated with the “mammoth fauna” in the Late Pleistocene localities of the Ural Mountains and West Siberia. ZooKeys.

[8] Gurina A.A., Dudko R.Y., Prosvirov A.S., Tshernyshev S.E., Legalov A.A., Zinovyev E.V. (2019). Coleoptera assemblages from the Quaternary deposits of Kizikha river, the southernmost late Pleistocene insects of the West Siberian Plain. Invertebr. Zool..

[9] Gurina A.A., Dudko R.Y., Ivanov A.V., Kotov A.A., Mikhailov Y.E., Prokin A.A., Prosvirov A.S., Solodovnikov A.Y., Zinovyev E.V., Legalov A.A. (2023). New Data on the Distribution of Southern Forests for theWest Siberian Plain during the Late Pleistocene: A Paleoentomological Approach. Diversity.

[10] Weather and Climate. http://www.pogodaiklimat.ru/climate.php.

[11] National Centers for Environmental Information. https://www.ncei.noaa.gov.

[12] Gurina A.A., Dudko R.Y., Zinovyev E.V., Legalov A.A. (2022). Quaternary paleoentomology: 10 years in the south of Western Siberia. Priroda.

[13] Mordkovich V.G., Dudko R.J., Khudyaev S.A., Lyubechanskii I.I. (2022). Changes in the Ground Beetle and Darkling Beetle Communities (Coleoptera: Carabidae, Tenebrionidae) in the Mountain Hollows of the Tuva and Altai Republics over 60 Years: A Trend or a Fluctuation?. Contemp. Probl. Ecol..

[14] Gurina A.A., Dudko R.Y., Zinovyev E.V., Borodin A.V., Tshernyshev S.E., Legalov A.A. (2018). Late Pleistocene taphocoenosis of insects and small mammals from the upper reaches of the Ob river. Paleontol. J..

[15] Dudko R.Y., Danukalova G.A., Gurina A.A., Ivanov A.V., Mikhailov Y.E., Osipova E.M., Prosvirov A.S., Solodovnikov A.Y., Legalov A.A., Zinovyev E.V. (2022). Insects and molluscs of the Late Pleistocene at the Gornovo site (Southern Ural foreland, Russia): New data on palaeoenvironment reconstructions. Quat. Int..

[16] Bakhmet’ev P.I. (1912). Theoretical and practical implications of my research on anabiosis in animals. Priroda.

[17] Salt R.W. (1936). Studies on the Freezing Process in Insects.

[18] Hou F., Ma J., Liu X., Wang Y., Liu X.N., Zhang F.C. (2010). Seasonal changes in antifreeze protein gene transcription and water content of beetle *Microdera punctipennis* (Coleoptera: Tenebrionidae) from Gurbantonggut desert in Central Asia. Cryoletters.

[19] Li N.G. (2011). Ice-nucleating activity of hemolymph of *Upis ceramboides* beetle inhabiting in central Yakutia. Probl. Cryobiol. Cryomed..

[20] Dudko R.Y., Alfimov A.V., Gurina A.A., Meshcheryakova E.N., Reshetnikov S.V., Legalov A.A., Berman D.I. (2024). Insufficient Cold Resistance as a Possible Reason for the Absence of Darkling Beetles (Coleoptera, Tenebrionidae) in Pleistocene Sediments of Siberia. Insects.

[21] Skopin N.G. (1974). Zur Revision der eurasiatischen Arten der Gattung *Belopus* Gb. Entomol. Abh..

[22] Iwan D., Löbl I. (2020). Catalogue of Palaearctic Coleoptera. Vol. 5, Tenebrionoidea.

[23] Kaszab Z. (1964). Beiträge zur Kenntnis der Tenebrioniden-Fauna des mittleren Teiles der Mongolischen Volksrepublik (Coleoptera). Acta zool. Acad. Sci. Hung..

[24] Medvedev G.S. (1990). Proceedings of the Zoological Institute. Vol. 220. Key to the Darkling Beetles of Mongolia.

[25] Kiselev S.V. (1973). Late Pleistocene Coleoptera of Transurals. Paleontol. J..

[26] Zinovyev E.V. (2020). Fauna Nasekomykh Urala i Zapadno-Sibirskoi Ravniny v Chetvertichnom Periode [Insect Fauna of the Ural and West Siberian Plain in the Quaternary Period]. Ph.D. Thesis.

[27] Gvozdetskii N.A., Mikhailov N.I. (1978). Fizicheskaya Geografia SSSR. Aziatskaya Chast’ [Physical Geography of the USSR. Asian Part].

[28] Strakhovenko V.D., Taran O.P., Ermolaeva N.I. (2014). Geochemical characteristics of the sapropel sediments of small lakes in the Ob’-Irtysh interfluve. Geol. Geophys..

[29] Berman D.I., Meshcheryakova E.N., Alfimov A.V., Leirikh A.N. (2002). The distribution of the earthworm *Dendrobaena octaedra* (Lumbricidae: Oligochaeta) in the north of the Holarctic is limited by insufficient frost resistance. Zool. Zhurn..

[30] Berman D.I., Leirikh A.N. (2019). Overwintering and Cold Hardiness of the Invertebrates in the Northeast Asia.

[31] RP5.ru Weather for 241 Countries of the World. https://rp5.ru/Weather_in_the_world.

[32] VNIIGMII-MCD All-Russian Research Institute of Hydrometeorological Information—World Data Center. http://www.meteo.ru/data.

[33] Pakhnev S.Y. (1965). Spravochnik po Klimatu SSSR [USSR Climate Guide.

[34] Polozova L.G. (1965). Spravochnik po Klimatu SSSR [USSR Climate Guide].

[35] Orlova V.V. (1966). Spravochnik po Klimatu SSSR [USSR Climate Guide].

[36] Orlova V.V. (1968). Spravochnik po Klimatu SSSR [USSR Climate Guide].

[37] Polozova L.G., Sharova V.Y. (1968). Spravochnik po Klimatu SSSR [USSR Climate Guide].

[38] Kukharskaya L.V. (1969). Spravochnik po Klimatu SSSR [USSR Climate Guide].

[39] Lebedev A.N., Kodrau O.D. (1974). Klimaticheskii Spravochnik Zarubezhnoi Azii. [Climate Reference Book of Foreign Asia].

[40] Pilnikova Z.N. (1993). Nauchno-Prikladnoi Spravochnik po Klimatu SSSR [Scientific and Applied Reference Book on the Climate of the USSR].

[41] Shulgin A.M. (1972). Klimat Pochv i Ego Regulirovanie [Soil Climate and Its Regulation].

[42] Nekrasov I.A. (1967). Taliki Rechnykh Dolin i Zakonomernosti ikh Rasprostraneniya [Taliks of River Valleys and Patterns of their Distribution].

[43] Voronina L.V. (1992). Teplovoi Rezhim Pochv Solontsovykh Kompleksov [Thermal Regime of Soils of Solonetz Complexes].

[44] Srebryanskaya N.I. (1954). Phenomena of seasonal freezing and thawing of soils of Central Baraba. Research of the Baraba Lowland as an object of agricultural use. Tr. Pochvennogo Instituta Im. V.V. Dokuchaeva.

[45] Velichko A.A. (2009). Paleoclimates and Paleoenvironmens of Extra-Tropical Regions of the Northern Hemisphere. Late Pleistocene—Holocene. Atlas-Monograph.

[46] Zykin V.S., Zykina V.S., Orlova L.A. (2003). Reconstruction of changes in the natural environment and climate of the Late Pleistocene based on sediments of Lake Aksor. Archaeol. Ethnol. Anthropol. Eurasia.

[47] Zykina V.S., Zykin V.S. (2012). Loess-Soil Sequence and Environment and Climate Evolution of West Siberia in Pleistocene.

[48] Izyumenko S.A. (1966). Spravochnik po Klimatu SSSR [USSR Climate Guide].

[49] Tsepelev K.A., Zinovyev E.V., Dudko R.Y., Tshernyshev S.E., Legalov A.A. (2013). Carrion beetles (Coleoptera, Silphidae) in younger Dryas of Chick River (late Pleistocene of Siberia). Euroasian Entomol. J..

[50] Zinovyev E.V., Dudko R.Y., Gurina A.A., Prokin A.A., Mikhailov Y.E., Tsepelev K.A., Tshernyshev S.E., Kireev M.S., Kostyunin A.E., Legalov A.A. (2016). First records of sub-fossil insects from Quaternary deposits in the southeastern part of West Siberia, Russia. Quat. Int..

